# Supplementary material for: Regulatory Interplay between miR-181a-5p and Estrogen Receptor Signaling Cascade in Breast Cancer
Source: Cancers (Basel). 2021 Feb 1;13(3):543. doi: 10.3390/cancers13030543 (PMC7867078; doi:10.3390/cancers13030543)
Supplement: Supplementary file 1 [file cancers-13-00543-s001.zip › cancers-1062600-supplementary figures.pdf]

cancers

Regulatory Interplay Between miR-181a-5p and Estrogen Receptor Signaling Cascade in Breast Cancer

Rosaria Benedetti, Chiara Papulino, Giulia Sgueglia, Ugo Chianese, Tommaso De Marchi, Francesco Iovino, Dante Rotili, Antonello Mai, Emma Niméus, Carmela Dell' Aversana and Lucia Altucci

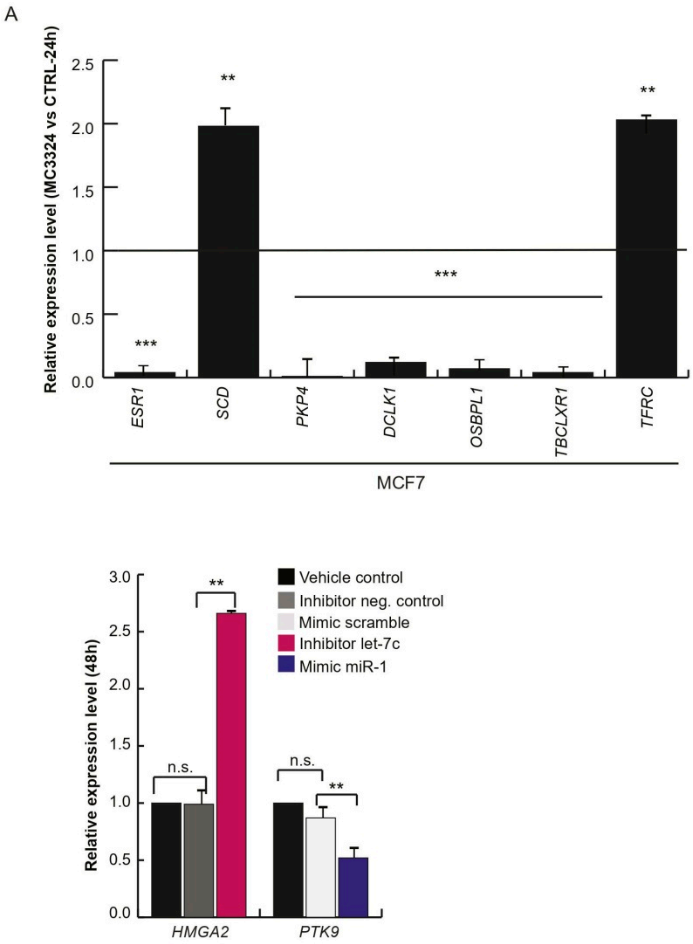

Figure S1. (A) Relative expression levels of selected miRNA targets as well as ERα and LSD1 protein interactors in MCF-7 cells after MC3324 treatment. (B) Relative expression levels of HMGA2 and PTK9 targets of miR-1 and let-7c positive controls, respectively, for miRNA mimic and inhibition approaches.

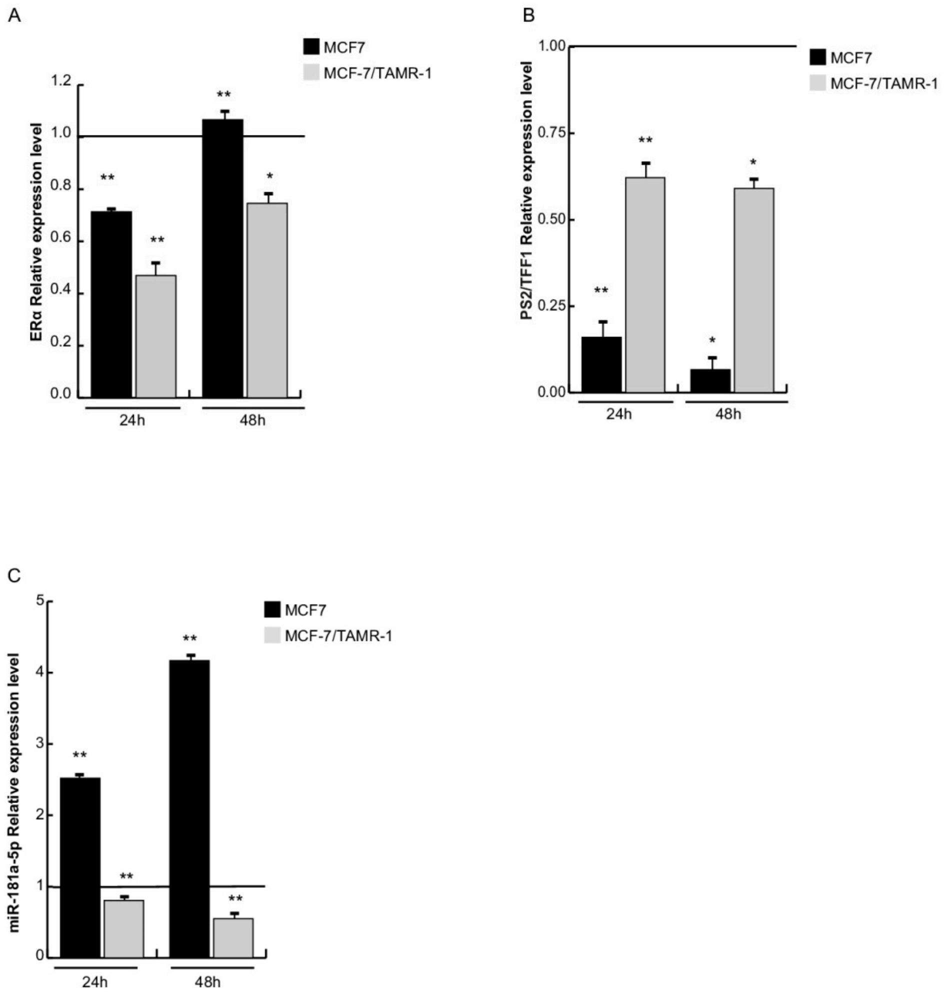

Figure S2. (A) ERα and PS2/TFF1 relative expression levels determined by qPCR after time dependent 4-OH-Tamoxifen treatment in MCF7 and MCF7/TamR1 cell lines. GAPDH was used for data normalization. (B) miR181a-5p relative expression level in MCF7 and MCF7/tamR1 cell lines following 24 and 48 h of 4-OH-Tamoxifen treatment. RNU6B was used for data normalization of miRNA expression. The results of three independent experiments each performed in triplicate are represented as the mean ± SD. ** $p \leq 0.01$; *** $p \leq 0.001$.

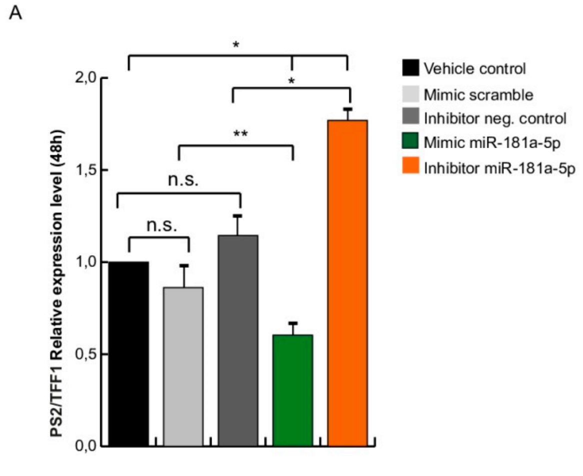

Figure S3. PS2/TFF1 relative expression levels determined by qPCR after transfection with synthetic mimic and inhibitor of miR-181a-5p and controls at a concentration of 100 nM for 48 h. GAPDH was used for data normalization. The results of three independent experiments each performed in triplicate are represented as the mean ± SD. ** $p \leq 0.01$; *** $p \leq 0.001$.
